# 

**Published:** 1921-07-16

**Authors:** 


### Priory Day in Wales.

An attempt is being made to organise throughout Wales and Monmouthshire a "Priory Day" towards the end of July, which it is hoped will be held annually, for the benefit of the work for ex-Service men undertaken by the Order of the Hospital of St. John of Jerusalem. A debit has to be cleared. The Priory is responsible for hospitals and hostels for ex-Service men. It is forming comforts depôts in industrial areas for those having home treatment, and for the sick poor where medical comforts are lent or obtainable for nominal sums. It is hoped, from the success of these depôts, to establish one in every town and village in Wales. The ambulance transport service is another useful but costly activity. A subscriber of a penny a week is entitled to call the car should illness or accident befall any member of his family.

### The General Nursing Council for Scotland.

At the Council Meeting, held at 13 Melville Street, Edinburgh, on June 29, the Registrar submitted a letter from the Scottish Board of Health desiring to know whether it is proposed that the Draft Syllabus prepared by the Council for Education and Training in General Nursing will apply also to fever nursing as to the first part thereof. After discussion the Council unanimously resolved that the first part of the Syllabus should apply also to the training of future nurses for the Fever Register and for the Children's Register. Correspondence in regard to the future training of mental nurses was referred to the Registration Committee.

The Registrar reported that he had not heard further from the Scottish Board of Health in regard to negotiations on the subject of nurses on the Board's existing Register of Fever Nurses, and it was arranged that the Chairman of the Council should endeavour to see the Board with a view to having matters expedited.

### Hospital Sunday, 1921.

The total amount received at the Mansion House to date is £70,000. Included in this total are the following collections:—St. Columba's Church of Scotland, Pont Street, £720; St. James's, Piccadilly, £428; Holy Trinity, Sloane Street, £304; St. Paul's, Knightsbridge, £265; St. Jude's, South Kensington, £244.

Mr. Arthur Jackson, of College Hill, Shrewsbury, for thirty years hon. surgeon to the Royal Salop Infirmary, has left estate valued at £13,522.

### The College of Nursing.

### (Limited by Guarantee.)

### LONDON CENTRE.

(Hon. Secretary, Miss Bompas, 7 Henrietta Street, W. 1.)

Tuesday, July 19, at 8 p.m.—The second "At Home" of the London Centre at No. 7 Henrietta Street. Members of King's College Hospital will act as hostesses. Music; recitation. Refreshments, 6d. All Centre members and their friends are invited.

### The Hospital

**RATES OF SUBSCRIPTION** (post free, payable in advance).

Three months, 3/3; six months, 6/6; twelve months, 13/-. (Should the cost of printing and paper as well as increased postage, necessitate alteration of these rates, the period paid for will be reduced proportionately on unexpired subscriptions.) **THE HOSPITAL "Index" to Volume LXIX., October 1920 to March 1921, is now ready, price 1/1 per copy, post free.** Address The Manager, The Hospital, "The Hospital" Building, 28/29 Southampton Street, Strand, London, W.C. 2.

